# Thinking through abrupt closure in humanitarian assistance: Key ethical considerations in seemingly impossible conditions

**DOI:** 10.1371/journal.pgph.0004656

**Published:** 2025-06-04

**Authors:** Lisa Eckenwiler, Isabel Munoz-Beaulieu, Revka Perez, Mayfourth Luneta, Shelley Rose Hyppolite, Lisa Schwartz, Matthew Hunt

**Affiliations:** 1 Department of Philosophy, George Mason University, Fairfax, Virginia, United States of America; 2 Department of Family Medicine, McGill University, Montréal Québec, Canada; 3 Center for Disaster Preparedness, Quezon City, Philippines; 4 Center for Disaster Preparedness, Quezon City, Philippines,; 5 Faculté de Médecine de l’Université Laval, Québec, and Centre Intégré Universitaire de Santé et de Services Sociaux de la Capitale-Nationale, Québec City, Canada,; 6 Department of Health Research Methods, Evidence, and Impact, McMaster University, Hamilton, Ontario, Canada,; 7 School of Physical and Occupational Therapy, McGill University, Montréal, Québec, Canada; PLOS: Public Library of Science, UNITED STATES OF AMERICA

USAID was the largest international development and humanitarian aid funder until it was abruptly halted on January 20^th^, 2025 – and later cancelled. Critical life-saving projects stopped overnight [1]. As a result, some health services disappeared, health outcomes are deteriorating, and survival is at risk [2,3].

We appreciate certain criticisms of existing models of humanitarian assistance and agree with calls for new ways forward [4–8]. Nevertheless, the sudden dismantling of USAID is not ethically defensible due to its direct, negative effects on global relations and valuable collaboration programs, and the immediate effects for people receiving assistance. For the humanitarian sector, this will be cascading: UN organizations, international, national and local NGOs, educational establishments, programs and projects (relating for example to vaccination, antiretroviral treatments for HIV patients, antimalaria efforts), were heavily reliant on USAID, and, as they falter or fail, civil society and humanitarian response capabilities will be undermined in many locales [9–11].

Our team conducts research on ethics and closure of humanitarian projects [12]. We explore questions of what it means to ‘close well’ as humanitarian organizations end a project. With a Guidance Note and a framework, “an ethics of the temporary”,[13,14] we offer resources on ethical responsibilities in closure. Our focus has been on planned project closures. We could not have anticipated circumstances of such widespread forced closures across the humanitarian sector. Yet, the resources we have developed can provide insight to those struggling to respond to the abrupt project closures resulting from the withdrawal of USAID funding. In this piece, we present four principles of “closing well” and illustrate how members from our research team at the Center for Disaster Preparedness Foundation, Inc. (CDP) are drawing upon them. CDP has been integral to planning, developing, implementing and disseminating the research underlying the development of the following principles: a) preserving relationships and promoting fairness, b) being transparent and accountable, c) preventing harms, and d) sustaining project gains and shifting toward localization (see Table 1). We will illustrate the application of these principles through discussing the recent experiences of the CDP. Our aim is to offer practical insights for humanitarian practice.

**Table 1:** Key ethical considerations for project closure in the context of USAID’s withdrawal.

Principles of “closing well”	Impact of USAID’s withdrawal on CDP and communities	Actions taken by CDP to uphold ethical conduct
Preserving relationships and promoting fairness	• Abrupt withdrawal limits the capacity and resources to honor relationships and respect appropriate closure procedures. • Without continued engagement, long-standing relationships may weaken, reducing future collaboration opportunities. • The potential for reduced equity and justice increases if local actors are not fully supported in the transition.	• CDP is honoring relationships and partnerships through continued communication.
Being transparent and accountable	• Confusion persists due to the lack of clarity from the funding agency, making it harder for the organization to plan. • Local organizations bear the burden of ensuring transparency and trust in the absence of clear guidance.	• The team has written to government and community partners and hosted face-to-face meetings to explain what they know and its immediate impact. • They are writing final reports to remain accountable to donors and partners despite uncertainty.
Preventing harms	• 1,467 disaster preparedness bags remain undistributed to high-risk families in the Agno River Basin which wastes resources, and reduces community preparedness, resilience and future ability to avert harm. • CDP has lost a portion of funds for operational activities, leading to some project staff being laid off.	• CDP secured partial staff funding to avoid immediate layoffs and support personnel through the transition.
Sustaining project gains and shifting towards localization	• Sudden loss of project funding jeopardizes long-term community benefits and progress toward equity. • Without continued organizational support, local actors may struggle to sustain DRR efforts and essential services. • Potential for setback in reducing global health and disaster risk inequities rises as communities face reduced resources.	• They are exploring new funding streams, training consultancies, local partnerships, and grant writing to maintain project benefits. • CDP is now more than ever employing an “asset-based” approach, focusing on local strengths (infrastructure, financial, political, social, and cultural capital). • Prioritization of partners for inclusion on capacity development programs will need to be supported by other funding sources.

CDP, a civil society organization in the Philippines, has been working around the Agno River Basin in the Philippines for the past 25 years. They had received USAID funds for a community-based disaster risk reduction and management (CBDRRM) project. The first phase focused on risk assessments and in 2025, preparedness activities for communities, flood response simulation exercises, and long-term planning for ecosystem-based adaptation were to be implemented. The project’s termination leaves hundreds of families vulnerable in high-risk flood zones with limited capacities to respond effectively when flooding occurs, thwarting adaptation.

## Preserving relationships and promoting fairness

Our work on ethics and project closure has emphasized community engagement for building trusting relationships, upholding ideals of procedural justice, and taking more informed action [13,14]. CDP has long prioritized community engagement and leadership. Ethical closure requires fair processes, with concern for equity in closure decisions *and* following exit. In recent weeks, CDP focused on identifying exit actions that do not require financial resources to honor relationships and support “closing well” even abruptly by, for example, maintaining communication with local partners. Through formal and continued open communication with government and community partners using online or instant messaging applications, CDP has sought to preserve relationships and demonstrate respect towards partners.

## Being transparent and accountable

Transparency is vital in humanitarian response activities and in international collaborations. It fosters trust and engagement, demonstrates respect for local actors and supports them to plan effectively. Despite the confusion and limited instruction from USAID, CDP is maintaining trust with its local partners through openness and honesty. They have written to government and community partners and hosted face-to-face meetings with available partners to explain what they know about the cuts and their immediate impacts. Amidst the sudden freeze of established contracts, local CDP responders remain accountable to staff and communities in these ways, as well as to USAID, by preparing their final report according to specifications.

## Preventing harms

Humanitarian organizations should seek to anticipate, prevent and mitigate harms that may occur during or due to their interventions. Some projects, like CDP’s, focus explicitly on averting future crises or lessening their effects. Other humanitarians are working on life-threatening disease or food security, for example. When projects are forced to close urgently under duress, harm – to health, finance, and often infrastructure – is inevitable and often severe. We have seen before that with abrupt closure, local and global health can be placed in peril, advances in reducing global inequities set back, and the potential for global health emergencies increased [15].

In the CDP project, emergency kits and other resources have gone undistributed in the Agno River Basin. Based on an assessment of its inventory, CDP found that 1,467 disaster preparedness bags packed with emergency supplies such as a blanket, flashlight, and first aid kits were intended to be provided to participants at the flood evacuation simulation drill. This includes hundreds of family level preparedness booklets, containing key information on disaster risk reduction (DRR), local early warning systems, emergency hotlines, and a guide for family disaster risk reduction planning that are now left in boxes. Discontinuing the flood simulation exercises may eliminate opportunities to practice and gain feedback on plans developed through local participatory processes, undermining capacity to avert and mitigate future harm, promote preparedness, build resilience, and (genuinely) wasting resources. At the organizational level, while CDP has lost a portion of funds for its operational expenses, it was able to secure funding to cover half of its staff salaries for a month avoiding immediate layoffs and the most imminent harms to staff and their families as they look for job opportunities. Fortunately, some staff found new jobs, while several others were re-assigned to new projects instead of hiring new personnel.

## Sustaining project gains and shifting towards localization

Even though humanitarian organizations are often focused on immediate crises, they also need to be concerned for the long-term well-being of communities and aim at sustaining projects’ gains to the extent possible. This approach supports preparedness and can advance local development. Despite the lack of time and resources to conduct turn-over ceremonies as would have normally accompanied the end of CDP’s project, copies of the finalized and formatted participatory disaster risk reduction plans and contingency plans formulated by the communities were provided to the local government. It is noteworthy that half have been recently adopted through council resolutions.

In many settings globally, local organizations continue to provide support to communities despite constraints imposed by the recent cuts.[9] All are likely to be seeking new funding streams. CDP has focused on building sustainable benefits, training consultancies, fundraising, selling merchandise, local partnerships, and grant writing. Anticipating the end of the 90-day stop work order, CDP has considered other means to pursue their work with local partners and government agencies and continue DRR awareness activities and the provision of disaster preparedness kits to families in the river basin. These partners are also prioritized and supported in other capacity development programs hosted by CDP. The organization fosters “asset-based approaches” by supporting natural, infrastructure, financial, political, social, cultural, and human capital of partner communities and engaging in capacity building (e.g., training “community facilitators” for participatory risk assessments and DRRM planning). CDP is committed to providing continued support, even if scaled back or reconfigured.

## Conclusion

While these are undoubtedly unprecedented times, and many local and international actors are facing seemingly impossible conditions, it is important to recognize that organizations like CDP are doing their best within extreme constraints. The abrupt nature of USAID’s funding withdrawal means that, in many cases, the conditions necessary for an ethical and well-planned closure—such as adequate time, resources, and preparation—are simply not available. Yet, the CDP example shows that even in the face of forced closure due to funding cuts, it is still possible for humanitarian organizations to uphold ethical principles and strive to safeguard the health and well-being of communities. It remains our collective responsibility to advocate for global health justice.
